# Prognostic value of cardiovascular magnetic resonance T1 mapping and extracellular volume fraction in nonischemic dilated cardiomyopathy

**DOI:** 10.1186/s12968-023-00919-y

**Published:** 2023-02-06

**Authors:** Farah Cadour, Morgane Quemeneur, Loic Biere, Erwan Donal, Zakarya Bentatou, Jean-Christophe Eicher, François Roubille, Alain Lalande, Roch Giorgi, Stanislas Rapacchi, Sébastien Cortaredona, Farouk Tradi, Axel Bartoli, Serge Willoteaux, François Delahaye, Stephanie M. Biene, Lionel Mangin, Nadine Ferrier, Jean-Nicolas Dacher, Fabrice Bauer, Guillaume Leurent, Pierre-Axel Lentz, Hélène Kovacsik, Pierre Croisille, Franck Thuny, Monique Bernard, Maxime Guye, Alain Furber, Gilbert Habib, Alexis Jacquier

**Affiliations:** 1 CNRS, CRMBM, Aix-Marseille University, Marseille, France; 2 CEMEREM, APHM, CHU Timone, Marseille, France; 3 Department of Cardiology, University Hospital of Angers, 49000 Angers, France; 4 UMR CNRS 6015-INSERMU1083, Institut Mitovasc, University of Angers, 49000 Angers, France; 5 Department of Cardiology, Inserm, LTSI–UMR 1099, CHU Rennes, Univ Rennes, 35000 Rennes, France; 6 Cardiology Department, University Hospital François Mitterrand, Dijon, France; 7 PhyMedExp, INSERM, CNRS, Cardiology Department, INI-CRT, CHU de Montpellier, Université de Montpellier, Montpellier, France; 8 ImViA Laboratory, University of Burgundy, 7 Bld Jeanne d’arc, 21000 Dijon, France; 9 Medical Imaging Department, University Hospital of Dijon, 21000 Dijon, France; 10 APHM, INSERM, IRD, SESSTIM, Sciences Economiques & Sociales de la Santé & Traitement de l’Information Médicale, ISSPAM, Hop Timone, BioSTICBiostatistique et Technologies de l’Information et de la Communication, Aix Marseille Univ, Marseille, France; 11 IRD, AP-HM, SSA, VITROME, Aix Marseille Univ, Marseille, France; 12 IHU-Méditerranée Infection, Marseille, France; 13 Department of Cardiology, Hospices civils de Lyon, 69002 Lyon, France; 14 Department of Radiology, CH d’Annecy, 74370 Annecy, France; 15 Department of Cardiology, CH d’Annecy, 74370 Annecy, France; 16 Department of Cardiology, CH de Vichy, 03207 Vichy, France; 17 Department of Radiology, UNIROUEN, Inserm U1096, CHU de Rouen, 76000 Rouen, France; 18 INSERM U 1096, Cardiovascular Surgery Department, Pulmonary Hypertension and Advanced Heart Failure Clinic, Rouen University Hospital, Rouen, France; 19 Department of Radiology, University Hospital Pontchaillou, Rennes, France; 20 Departement of Cardiovascular Imaging, Chu Montpellier, Montpellier, France; 21 Department of Radiology, University Hospital Saint-Etienne, Saint-Étienne, France; 22 CNRS UMR 5520, INSERM U1294, CREATIS, INSA-Lyon, Univ Lyon, UJM-Saint-Etienne, 42023 Saint-Étienne, France; 23 Unit of Heart Failure and Valvular Heart Diseases, Inserm 1263, Inrae 1260, Department of Cardiology, North Hospital, Assistance Publique - Hôpitaux de Marseille, Centre for CardioVascular and Nutrition Research (C2VN), University Mediterranean Center of Cardio-Oncology, Aix-Marseille University, Marseille, France; 24 Cardiology Department, IRD, APHM, MEPHI, IHU-Méditerranée Infection, APHM, La Timone Hospital, Aix Marseille Univ, Marseille, France; 25 Faculté de Médecine, CNRS, Aix-Marseille Université, 27 Bd Jean Moulin, 13385 Marseille cedex 5, France

**Keywords:** Nonischemic dilated cardiomyopathy, Cardiac magnetic resonance, Extracellular volume (ECV), Native T1, Late gadolinium enhancement, Prognostic value, Myocardial fibrosis

## Abstract

**Background:**

Heart failure- (HF) and arrhythmia-related complications are the main causes of morbidity and mortality in patients with nonischemic dilated cardiomyopathy (NIDCM). Cardiovascular magnetic resonance (CMR) imaging is a noninvasive tool for risk stratification based on fibrosis assessment. Diffuse interstitial fibrosis in NIDCM may be a limitation for fibrosis assessment through late gadolinium enhancement (LGE), which might be overcome through quantitative T1 and extracellular volume (ECV) assessment. T1 and ECV prognostic value for arrhythmia-related events remain poorly investigated. We asked whether T1 and ECV have a prognostic value in NIDCM patients.

**Methods:**

This prospective multicenter study analyzed 225 patients with NIDCM confirmed by CMR who were followed up for 2 years. CMR evaluation included LGE, native T1 mapping and ECV values. The primary endpoint was the occurrence of a major adverse cardiovascular event (MACE) which was divided in two groups: HF-related events and arrhythmia-related events. Optimal cutoffs for prediction of MACE occurrence were calculated for all CMR quantitative values.

**Results:**

Fifty-eight patients (26%) developed a MACE during follow-up, 42 patients (19%) with HF-related events and 16 patients (7%) arrhythmia-related events. T1 Z-score (p = 0.008) and global ECV (p = 0.001) were associated with HF-related events occurrence, in addition to left ventricular ejection fraction (p < 0.001). ECV > 32.1% (optimal cutoff) remained the only CMR independent predictor of HF-related events occurrence (HR 2.15 [1.14–4.07], p = 0.018). In the arrhythmia-related events group, patients had increased native T1 Z-score and ECV values, with both T1 Z-score > 4.2 and ECV > 30.5% (optimal cutoffs) being independent predictors of arrhythmia-related events occurrence (respectively, HR 2.86 [1.06–7.68], p = 0.037 and HR 2.72 [1.01–7.36], p = 0.049).

**Conclusions:**

ECV was the sole independent predictive factor for both HF- and arrhythmia-related events in NIDCM patients. Native T1 was also an independent predictor in arrhythmia-related events occurrence. The addition of ECV and more importantly native T1 in the decision-making algorithm may improve arrhythmia risk stratification in NIDCM patients.

*Trial registration* NCT02352129. Registered 2nd February 2015—Retrospectively registered, https://clinicaltrials.gov/ct2/show/NCT02352129

**Supplementary Information:**

The online version contains supplementary material available at 10.1186/s12968-023-00919-y.

## Background

Nonischemic dilated cardiomyopathy (NIDCM) is a public health concern with a prevalence ranging from 1/400 to 1/250 in the general population [1]. Dilated cardiomyopathy (DCM) is defined as left ventricular (LV) dilatation with systolic dysfunction and may be either idiopathic or secondary to multiple causes. Even if rhythm disorders are common [2], heart failure (HF)-related events are the prevailing cause of morbidity and mortality in patients with NIDCM [3]. Current therapeutic guidelines are based upon LV ejection fraction (LVEF) and clinical symptoms to answer both HF- and arrhythmia-related complications [4]. Hence, implantable cardioverter-defibrillators (ICDs) are recommended for primary prevention in patients with symptomatic HF (New York Heart Association (NYHA) class II-III) and LVEF ≤ 35% despite 3 months of optimal medical treatment and for secondary prevention in individuals with ventricular arrhythmia. Risk stratification in NIDCM patients should deal with both HF-related events and arrhythmia-related events. Treatment options for HF and arrhythmia-related events are different, thus indicating the need to improve risk stratification of NIDCM patients and prognosis evaluation [5]. Interestingly, cardiovascular magnetic resonance (CMR) has emerged as a relevant tool in risk stratification, in addition to provide information on the possible underlying etiology of NIDCM patients [6, 7]. CMR commonly identifies focal replacement fibrosis using late gadolinium enhancement (LGE), but NIDCM is also associated with increased interstitial fibrosis which cannot be evaluated by LGE [8]. More recent quantitative CMR techniques, particularly myocardial T1 mapping, have emerged as novel methods for diffuse interstitial fibrosis assessment [9, 10]. Indeed, T1 mapping performed prior to and after gadolinium injection can provide an estimate for extracellular volume fraction (ECV), which is a quantitative marker of interstitial contrast agent accumulation [11]. Most studies evaluating T1 mapping parameters were monocentric, based on a single CMR scan, or more focused on HF-related events [8, 12–14]. Puntmann et al. [8] assessed the prognostic value of T1 mapping parameters in a large multicentric, without special regard of arrhythmic endpoints despite their importance in NIDCM.

The objective of our study was therefore to evaluate the prognostic value of CMR findings, including quantitative T1 and ECV, for both HF-related and arrhythmia-related events in NIDCM patients.

## Methods

### Study design

This was a prospective longitudinal multicenter study in which a cohort of adult patients with NIDCM was followed for 2 years. All 15 participating French centers were referral university hospital centers specialized in CMR. The study was approved by the institutional ethics committees, and written informed consent was obtained from all participants (NCT02352129). All procedures were carried out in accordance with the Declaration of Helsinki.

A total of 262 consecutive adults meeting the diagnostic criteria for NIDCM [15, 16] were enrolled between December 2011 and January 2017. Patients meeting the following inclusion criteria for DCM were eligible: typical symptoms of HF at the time of diagnosis and an LVEF < 45% with a LV end-diastolic volume (LVEDV) > 90 ml/m^2^ measured by echocardiography. Patients were excluded if DCM was caused by hypertension, ischemic or valvular disease or hypertrophic cardiomyopathy based on previous medical history or CMR findings. Additional exclusion criteria were generally accepted contraindications to CMR (claustrophobia, implantable devices, former metallic cardiac valves and non-CMR compatible vascular clips) or a history of renal disease with a current estimated glomerular filtration rate < 30 ml/min/1.73 m^2^. Patients with hepatic insufficiency; bone metabolism abnormalities, which influence the fibrosis process; or unstable, nontreated or acute HF during the past month were also excluded. Demographics, medical history, NYHA class, medications and laboratory tests were collected for all subjects.

### Study procedures

All subjects underwent CMR on a 1.5 T or 3 T scanner (Additional file 1: Table S1) in addition to clinical examination, electrocardiogram (ECG), rhythmic 24 h-holter and echocardiography at baseline. Applied CMR protocols compiled with local institutional practices to match with daily clinical practices, but were similar in the key elements of the protocol (Additional file 1: Table S2). Assessment of cardiac volume, mass and LVEF were performed on contiguous short-axis slices from base to apex. Cine CMR images were acquired in long-axis views (2- and 4-chambers and LV outflow tract) with an ECG-gated balanced steady-state free precession sequence. To match daily clinical practices, LGE sequence choice was left to the local teams but was performed at 10 min after injection by an inversion-recovery gradient echo or phase sensitive inversion recovery gradient echo sequence in three different planes (short-axis, 2- and 4-chambers). T1 mapping using a Modified Look-Locker Imaging technique (MOLLI) with embedded motion correction was performed before and at 15 min after intravenous injection of 0.2 mmol/kg gadoterate meglumine (Dotarem®, Guerbet, France). For 1.5 and 3 T studies, a 5(3)3 and a 4(1)3(1)2 MOLLI acquisition schemes were respectively used for pre and postcontrast T1 mapping [17, 18] and performed over three slices in the LV short-axis view (base, mid, apex).

### Image postprocessing and analysis

All images were independently analyzed by two radiologists specialized in cardiac imaging (M.Q. and A.J.) who were blinded to the clinical data. Quantification of LV volumes and function and analysis of LGE were performed centrally with Argus software (Siemens Healthineers, Erlangen, Germany). The presence of LGE was visually assessed, by consensus agreement of the two readers in case of discrepancies, and defined as linear midwall enhancement visible on two different views with one of the two being the short-axis view.

**Fig. 1:**
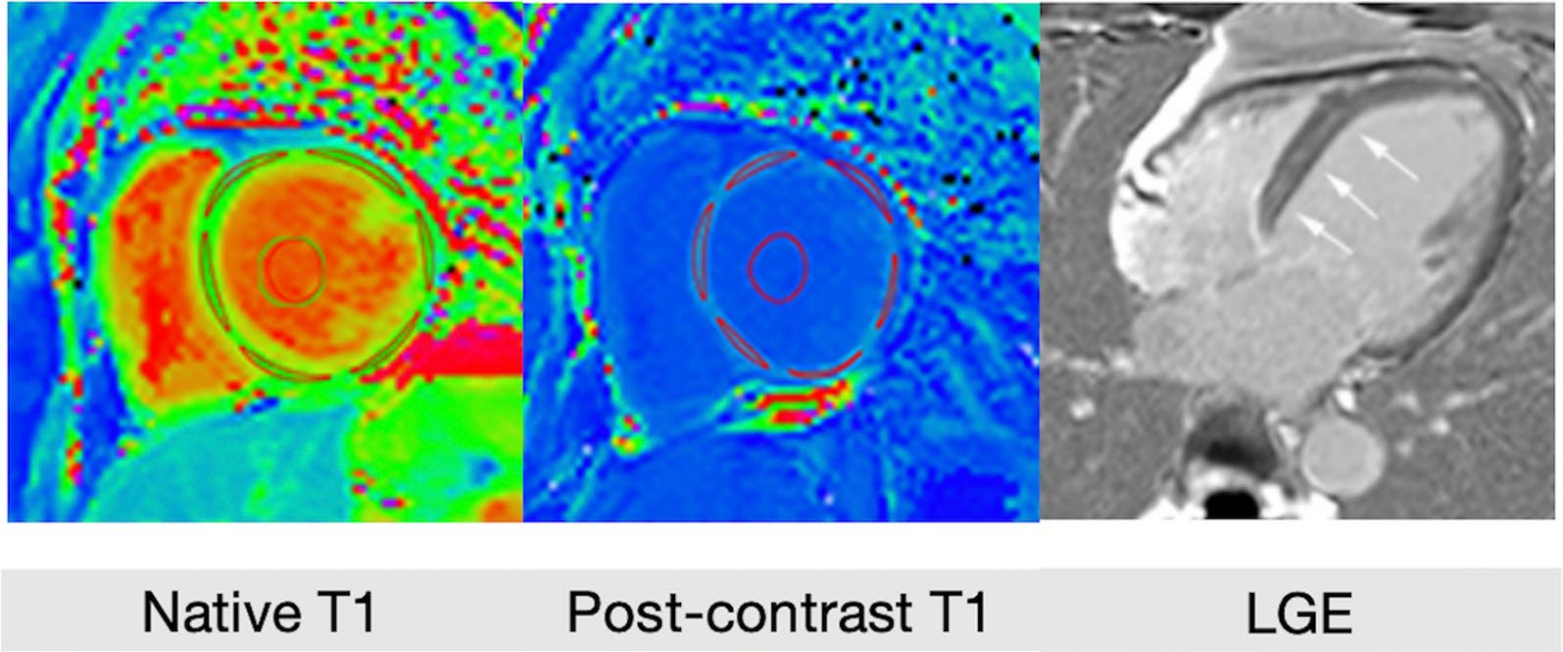
Image analysis and post-processing. Segmentation of both native T1 and post-contrast T1 myocardium and blood pool allowed Extracellular volume fraction (ECV) quantification, in the 16 segments of the myocardium. Linear midwall enhancement in late gadolinium enhancement (LGE) enabled identification of focal fibrosis (arrows)

Pre- and postcontrast T1 maps were analyzed using OsiriX software (Pixmeo, Geneva, Switzerland). ECV was measured directly on the ECV map and was calculated by the software from pre- and post-T1 maps and the patient’s hematocrit value [19] (Fig. 1). ECV measures were carried out in the 16 segments at the basal, mid and apical myocardial levels. The subendocardial and subepicardial regions were excluded (offset values, 5%) to avoid partial volume effects with blood or epicardial fat [17]. The ECV global value was calculated as the segmental mean value of the 16 segments for each patient. ECV global values of the base, mid and apex slices were calculated as the segmental mean values on the corresponding segments (base: segments 1–6; mid: 7–12; apex: 13–16). The maximum ECV among all segments was also reported. Patient’s hematocrit value was derived from routine blood tests performed the same day as the CMR exam [20]. Myocardial fibrosis was distinguished between focal replacement fibrosis caused by myocardial infarction and diffuse interstitial fibrosis, characterized by the accumulation of collagen in myocardial interstitial tissue. In the present study, we considered the presence of LGE to be a surrogate marker of focal replacement fibrosis; and an increased native myocardial T1 and/or myocardial ECV to be a surrogate marker of diffuse interstitial fibrosis [21]. To enable combined analysis of different CMR scanners, T1 values were converted to Z-scores.

### Outcomes

The primary outcome was long-term prognosis, assessed according to major adverse cardiovascular events (MACE) occurrence. MACEs were divided into two groups: [1] HF-related events, including: HF death, HF hospitalizations, heart transplant, LV assist device implantation for advanced HF; and [2] arrhythmia-related events, including: sudden death (SD), sustained ventricular tachycardia (VT), appropriate ICD shocks, resuscitated cardiac arrest, ventricular fibrillation. When more than one event occurred in a patient, the first event was used. The outcome data were collected during 2 years of follow-up through electronic medical records and systematic phone calls every 6 months by an independent physician blinded to the imaging results. Cardiac cause of death was verified by death certificates and medical records. VT was identified on an ECG in the case of symptomatic patients and by recording of an arrhythmia if the patient had an ICD or rhythmic holter.

### Statistical analysis

Continuous variables were expressed as the mean ± standard deviation (SD) or as the median and interquartile range, depending on the normality of the distribution. Categorical variables were presented as the number of patients and percentages. Continuous variables were compared using Student’s t-test or the Mann–Whitney test, and categorical variables using the chi-square test or Fisher’s exact test as appropriate. MACE-free survival curves were generated using the Kaplan–Meier method. Time to event was measured from the date of first CMR. Univariate and multivariate associations of risk covariates with MACEs were determined by logistic and Cox proportional hazards regression. For each outcome (MACE, HF-related events and arrhythmia-related events), any statistically significant factor in univariate analyses (at p < 0.10) was selected as a potential candidate for the multivariate analysis. Multivariate Cox proportional hazards models were performed with a stepwise selection (likelihood ratio, significance level for entry: p = 0.10, significance level for staying in the model: p = 0.05) modeling to determine independent associations with the outcomes (adjusted hazard ratio, HR, and 95% confidence interval). A receiver operating characteristic curve analysis was used to identify the optimal LVEF, T1 Z-score, and ECV value to discriminate patients with and without a risk of HF- or arrhythmia-related events. The optimal cutoff point was calculated by determining the value that provided the best sensitivity and specificity based on the Youden index. These cutoff values were used for the Kaplan–Meier curves and the Cox regressions.

All analyses considered two-sided p-values, with statistical significance defined by p ≤ 0.05. Statistical analyses were performed with SAS (version 9.4, SAS Institute, Cary, North Carolina, USA).

## Results

### Study population

**Fig. 2:**
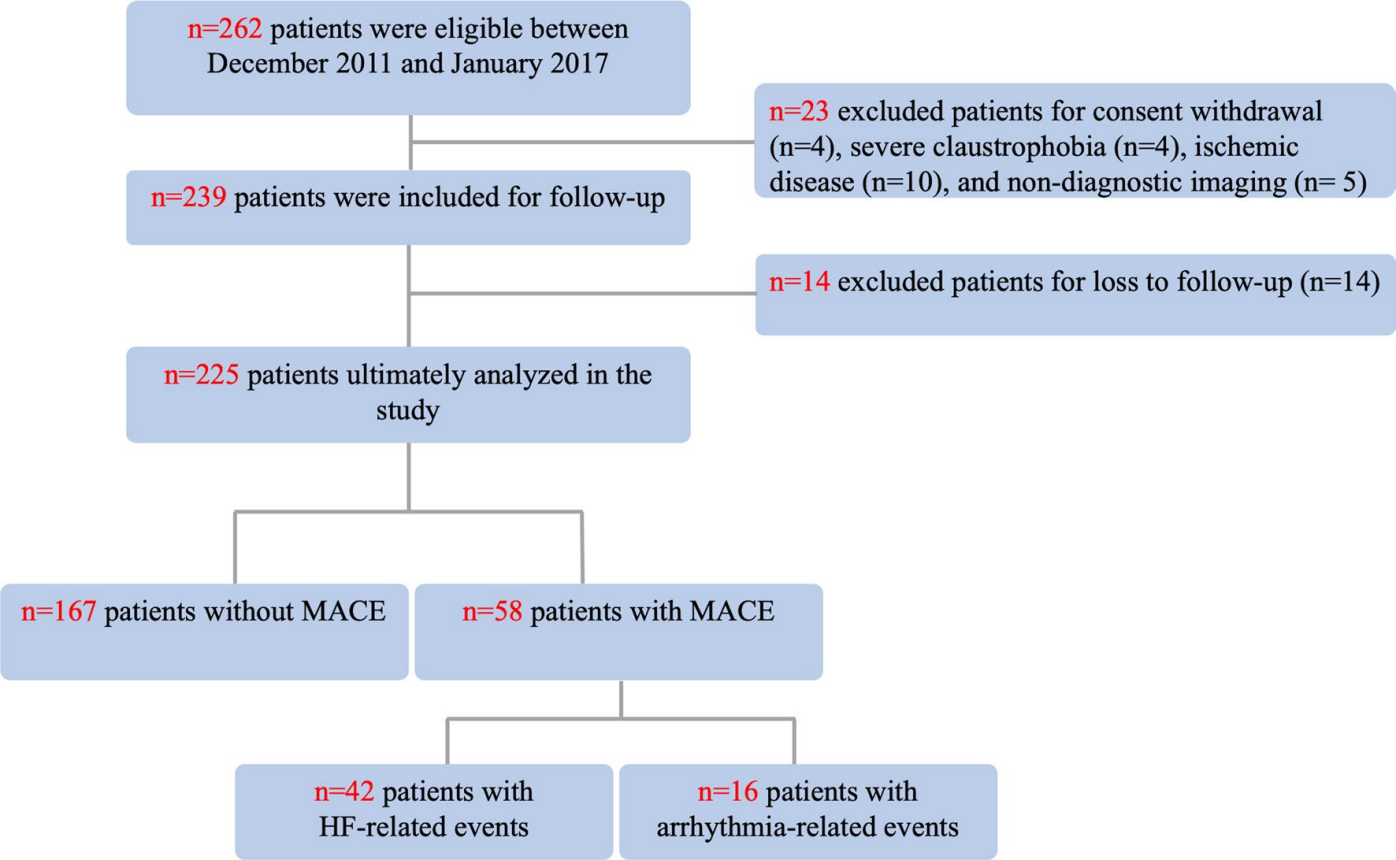
Flow chart of the study protocol.* HF* heart failure, *MACEs* major adverse cardiovascular events

Among the 262 consecutive patients included in the study, 37 patients (14%) were excluded from the final analysis due to loss to follow-up (n = 14), withdrawal of consent (n = 4), severe claustrophobia during CMR (n = 4), ischemic disease (n = 10) or nondiagnostic imaging due to artifacts (n = 5). The remaining 225 subjects constituted the study cohort (Fig. 2). The baseline characteristics of the patients are summarized in Table 1. The mean age of the cohort was 57 ± 14 years, and 65% of subjects were men. A total of 147 (81%) patients had a NYHA functional class of II or less. The mean LVEF and LVEDV were 29.3 ± 9.7% and 145 ± 48 ml/m^2^, respectively. LGE was present in 52% of the patients, and the mean native T1 Z-score and ECV were respectively 3.0 ± 2.3 and 29.3 ± 4.1%.

**Table 1:** Patients’ baseline characteristics (n = 225)

	All patients (n = 225)	No HF- nor arrhythmia-related events “No MACE” (n = 167)	HF-related events (n = 42)		Arrhythmia-related events (n = 16)	
				p-value^*^		p-value^*^
Demographics						
Age, years	57.5 ± 14.5	58.3 ± 13.7	53.5 ± 16.9	0.115	60.6 ± 15.1	0.455
Male, %	146 (64.9)	100 (61.7)	34 (81.0)	**0.028**	12 (75.0)	0.418
Clinical and biological indices						
Heart rate, beats/min (nmiss = 49)	73 ± 16	72 ± 16	77 ± 18	0.051	74 ± 15	0.469
Systolic blood pressure, mmHg (nmiss = 43)	123 ± 23	124 ± 22	116 ± 19	**0.012**	124 ± 35	0.377
Diastolic blood pressure, mmHg (nmiss = 43)	74 ± 14	74 ± 13	72 ± 14	0.281	75 ± 25	0.275
NYHA functional class (nmiss = 43)						
≤ II	147 (80.8)	115 (86.5)	22 (61.1)	**0.001**	10 (76.9)	0.401
> II	35 (19.2)	18 (13.5)	14 (38.9)	**0.001**	3 (23.1)	0.401
eGFR, ml/mn (nmiss = 32)	89 ± 40	87 ± 37	99 ± 47	0.179	87 ± 40	0.954
Hematocrit, % (nmiss = 34)	41.9 ± 4.5	41.5 ± 4.4	42.4 ± 4.3	0.322	44.1 ± 5.1	0.101
Cardiovascular risk factors						
Body mass index, kg/m^2^ (nmiss = 31)	26.7 ± 5.2	26.8 ± 5.2	26.8 ± 5.4	0.921	25.2 ± 5.0	0.343
Hypertension (nmiss = 28)	66 (33.5)	52 (35.9)	10 (25.6)	0.258	4 (30.8)	1.000
Diabetes mellitus (nmiss = 28)	29 (14.7)	19 (13.10)	8 (20.5)	0.306	2 (15.4)	0.684
Atrial fibrillation (nmiss = 28)	13 (6.6)	6 (4.1)	4 (10.3)	0.223	3 (23.1)	**0.028**
Dyslipidemia (nmiss = 28)	52 (26.4)	40 (27.6)	9 (23.1)	0.685	3 (23.1)	1.000
Smoking, current or previous (nmiss = 28)	76 (38.6)	55 (37.9)	16 (41.0)	0.716	5 (38.5)	1.000
Alcohol excess (nmiss = 30)	14 (7.2)	10 (7.0)	4 (10.3)	0.503	0 (0.0)	1.000
Family history of cardiomyopathy or SD (nmiss = 29)	32 (16.3)	24 (16.7)	6 (15.4)	1.000	2 (15.4)	1.000
Medication						
RAS inhibitors (nmiss = 25)	186 (93.0)	140 (95.2)	33 (54.6)	**0.032**	13 (92.9)	0.525
Diuretics (nmiss = 26)	151 (75.9)	107 (73.3)	32 (82.1)	0.303	12 (85.7)	0.522
Beta blocker (nmiss = 26)	183 (92.0)	136 (93.2)	35 (89.7)	0.498	12 (85.7)	0.282
CMR parameters						
LVEF, % (nmiss = 3)	29.3 ± 9.7	30.7 ± 9.7	24.4 ± 9.0	**< 0.001**	28.0 ± 6.58	0.267
LVEDV, ml/m^2^ (nmiss = 7)	145 ± 48	141 ± 47	159 ± 46	**0.011**	150 ± 54	0.423
LV mass, g/m^2^ (nmiss = 10)	90.5 ± 24.4	90.3 ± 25.2	91.7 ± 21.8	0.334	88.9 ± 23.5	0.858
LGE presence, % (nmiss = 7)	113 (51.8)	76 (47.2)	26 (63.4)	0.080	11 (68.8)	0.120
Native T1 Z-score (nmiss = 8)	3.0 ± 2.3	2.7 ± 2.2	3.8 ± 2.5	**0.008**	4.0 ± 1.6	**0.014**
ECV, % (nmiss = 12)	29.3 ± 4.1	28.7 ± 3.8	31.3 ± 4.5	**0.002**	30.6 ± 3.8	0.057
Global strain (nmiss = 29)	− 8.8 ± 2.9	− 9.1 ± 2.9	− 7.4 ± 2.9	**< 0.001**	− 9.1 ± 2.1	0.908

Bold values indicate significant p value (p ≤ 0.05)

^*^Fisher’s exact test for qualitative variables and Wilcoxon’s test for quantitative variables. Reference group in “No MACE” (n = 167). Values are mean ± standard deviation (SD), n (%). P ≤ 0.05 (versus patients without MACEs) is considered significant

*HF* heart-failure, *MACE* major adverse cardiovascular event, *NYHA* New York Heart Association, *eGFR* estimated glomerular filtration rate, *BMI* body mass index, *SD* sudden death, *RAS* renin–angiotensin–aldosterone system, *CMR* cardiac magnetic resonance, *LVEF* left ventricular ejection fraction, *LVEDV* left ventricular end-diastolic volume, *LV* left ventricular, *LGE* late gadolinium enhancement, *ECV* extracellular volume fraction, *nmiss* number of missing values

During a median follow-up of 23.9 (18.7–24.2) months, a total of 58 patients (26%) developed MACEs (Additional file 1: Table S3). HF-related events were the principal MACE (n = 42, 19%). 9 patients died (4%): 6 patients from HF death (3 from cardiogenic shock and 3 from refractory acute pulmonary edema) and 3 patients from SD (Additional file 1: Table S4). The remaining patients presented during follow-up either hospitalizations for HF (n = 29), heart transplant (n = 7), sustained VT (n = 8), or appropriate ICD shocks (n = 5).

### Prognostic value of CMR findings for HF-related events

In the HF-related events group, patients with a higher NYHA class were more prone to developing a MACE (p = 0.001). According to univariate analysis, patients with HF-related events had worse LVEF (24.4 ± 9.0 vs. 30.7 ± 9.7%; p < 0.001), increased LVEDV (159 ± 46 vs. 141 ± 47 ml/m^2^; p = 0.011) (Table 1). Patients who presented HF-related events showed significantly higher values of ECV and T1 Z-score (31.3 ± 4.5% vs 28.7 ± 3.8%; p = 0.002 and 3.8 ± 2.5 vs 2.7 ± 2.2; p = 0.008, respectively). These parameters were also found to be predictive of HF-related events development, with an increase of 1% of the global ECV and native T1 Z-score leading respectively to a + 17% and + 22% higher risk (OR 1.17 [1.07–1.28], p = 0.001, AUC 0.66 and OR 1.22 [1.04–1.42], p = 0.014, AUC 0.64, respectively) (Table 2). The optimal ECV cutoff value was 32.1%, with an almost fourfold increase in risk when the value was above this threshold (OR 3.56 [1.66–7.64], p = 0.001, AUC 0.62).

**Table 2:** CMR parameters—univariate analysis in the prediction of HF- and arrhythmia-related events

	HF-related events (n = 42)				Arrhythmia-related events (n = 16)			
	OR	95% CI^a^	p-value^*^	AUC (%)^b^	OR	95% CI^a^	p-value^*^	AUC (%)^b^
HR	1.02	1.00–1.04	0.087	60.8	1.01	0.98–1.05	0.587	56.1
LVEF	0.93	0.89–0.97	**< 0.001**	69.8	0.97	0.92–1.03	0.281	58.5
LVEF > optimal cutoff^c^	0.21	0.10–0.45	**< 0.001**	68.3	0.42	0.12–1.54	0.191	58.3
LVEDV	1.01	1.00–1.01	**0.032**	63.0	1.00	0.99–1.01	0.479	56.1
LV mass	1.00	0.99–1.02	0.728	54.9	1.00	0.98–1.02	0.830	48.6
LGE presence	1.94	0.96–3.93	0.066	58.1	2.46	0.82–7.40	0.109	60.8
Native T1 Z-score	1.22	1.04–1.42	**0.014**	64.0	1.27	1.02–1.59	**0.035**	68.9
Native T1 Z-score > optimal cutoff^d^	3.00	1.46–6.20	**0.003**	62.8	3.41	1.20–9.70	**0.022**	63.7
ECV global	1.17	1.07–1.28	**0.001**	66.1	1.13	0.99–1.29	0.070	65.0
Base ECV	1.11	1.02–1.20	**0.011**	61.9	1.08	0.96–1.22	0.206	59.0
Mid-ECV	1.14	1.05–1.23	**0.001**	65.7	1.05	0.93–1.19	0.409	57.4
Apex ECV	1.07	0.99–1.16	0.077	55.9	1.12	0.99–1.26	0.076	65.4
ECV > optimal cutoff^e^	3.56	1.66–7.64	**0.001**	62.3	3.58	1.21–10.61	**0.022**	65.2
ECV maximum	1.10	1.04–1.17	**0.002**	62.9	1.07	0.98–1.18	0.139	61.5

Bold values indicate significant p value (p ≤ 0.05)

*HF* heart failure, *CMR* cardiac magnetic resonance imaging, *HR* heart rate, *LVEF* left ventricular ejection fraction, *LVEDV* left ventricular end-diastolic volume, *LV* left ventricular, *NYHA* New York Heart Association, *LGE* late gadolinium enhancement, *ECV* extracellular volume fraction

*P ≤ 0.05 is considered significant

^a^Odds ratios with 95% confidence intervals (univariate logistic regression)

^b^Area under the ROC curve

^c^The Youden index was used to depict optimal cutoff values from the ROC curves (27.8 for MACE, 27.8 for heart failure and 34.0 for arrhythmia)

^d^The Youden index was used to depict optimal cutoff values from the ROC curves (4.0 for MACE, 3.8 for heart failure and 4.2 for arrhythmia)

^e^The Youden index was used to depict optimal cutoff values from the ROC curves (30.1 for MACE, 32.1 for heart failure and 30.5 for arrhythmia)

**Fig. 3:**
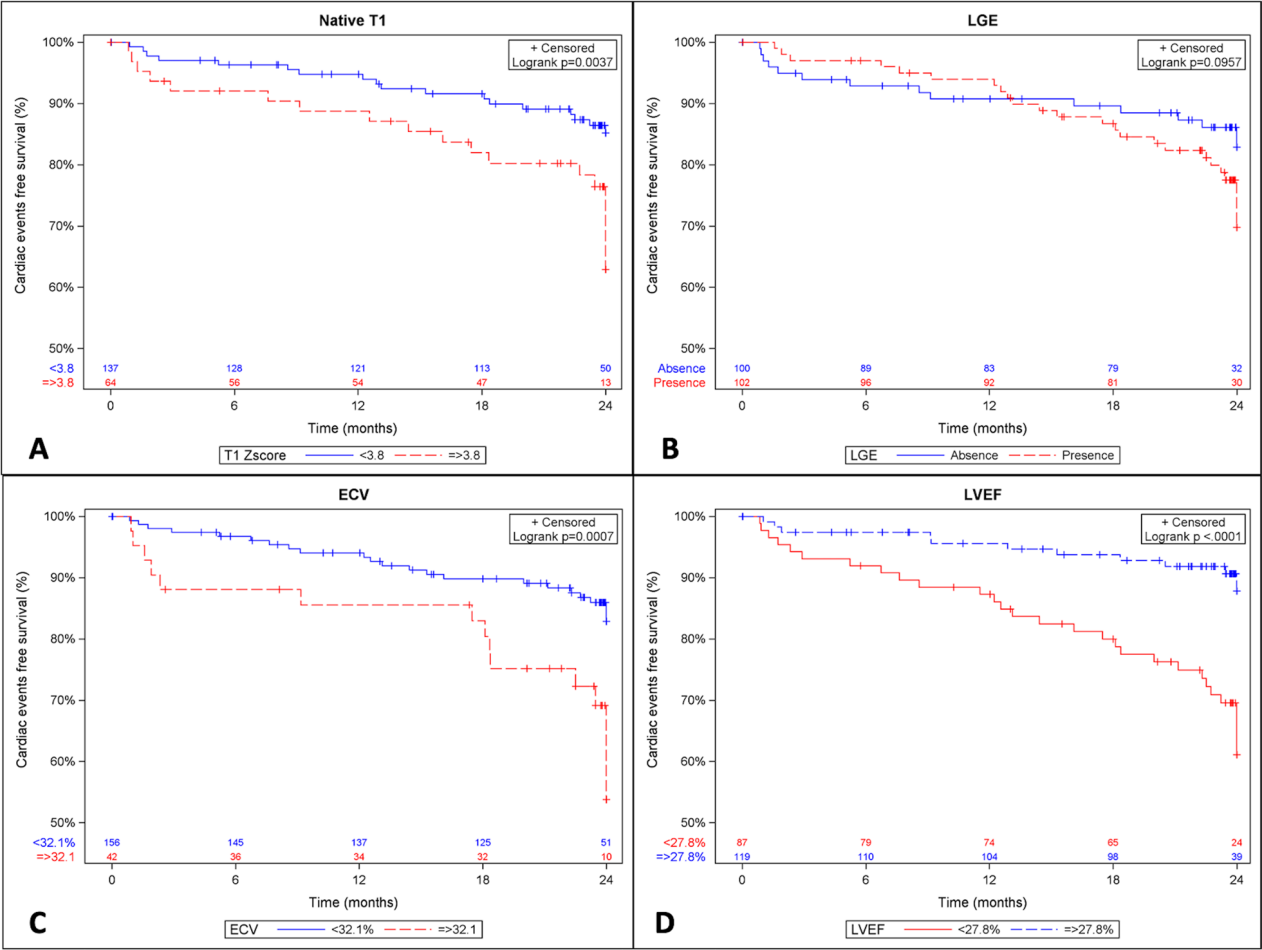
Cumulative 2-year heart failure (HF)-related events-free survival rate depending on CMR parameters. **A** Native T1 Z-score: < 3.8 vs. ≥ 3.8; based on the optimal cutoff determined by the Youden index. **B** Late gadolinium enhancement (LGE): present vs. absent. **C** Extracellular volume (ECV): < 32.1% vs. ≥ 32.1%; based on the optimal cutoff determined by the Youden index. **D** Left ventricular ejection fraction (LVEF): < 27.8% vs. ≥ 27.8%; based on the optimal cutoff determined by the Youden index

Kaplan–Meier curves showed that patients with LVEF < 27.8%, native T1 Z-score ≥ 3.8, and ECV ≥ 32.1% were at higher risk of HF-related events occurrence (p < 0.001, p = 0.004, and p < 0.001, respectively) (Fig. 3).

Multivariate analyses showed that, ECV was the only CMR independent predictor of HF-related events occurrence (HR 2.15 [1.14–4.07], p = 0.018) when above the optimal 32.1% threshold (Table 3). Sex, NYHA, and LVEF were also significantly associated with the prediction of HF-related events (p = 0.038, p = 0.002, and p = 0.008, respectively).

**Table 3:** Multivariate analysis of HF- and arrhythmia-related events prediction—stepwise Cox proportional hazards regression

Variable	Adjusted hazard-ratio	95% CI		p-value^*^
Outcome: HF-related events (n = 209)				
Men (ref. Women)	2.28	1.05	4.96	**0.038**
NYHA2 > II (ref. ≤ II)	2.82	1.45	5.49	**0.002**
LVEF > 27.8^a^	0.38	0.19	0.77	**0.008**
ECV > 32.1^a^	2.15	1.14	4.07	**0.018**
Outcome: arrhythmia-related events (n = 183)				
Atrial fibrillation (ref. no)	5.05	1.43	17.88	**0.012**
T1 Z-score > 4.2^a^	2.86	1.06	7.68	**0.037**
ECV > 30.5^a^	2.72	1.01	7.36	**0.049**

Bold values indicate significant p value (p ≤ 0.05)

*HF* heart failure, *LVEF* left ventricular ejection fraction, *LVEDV* left ventricular end-diastolic volume, *LV* left ventricular, *NYHA* New York Heart Association, *LGE* late gadolinium enhancement, *ECV* extracellular volume fraction

^*^P ≤ 0.05 is considered significant

^a^For each outcome, the Youden index was used to depict optimal cutoff values from the ROC curves (see Table 2)

Multivariate analyses in the HF-related events group were similar to those made for exploratory purposes on all MACE (HF- or arrhythmia-related events) (Additional file 1: Tables S5, S6, and Fig. S1).

### Prognostic value of CMR findings for arrhythmia-related events

Sixteen (7%) patients developed arrhythmia-related events during follow-up. Native T1 Z-score and ECV > 30.5% (optimal cutoff for patients with arrhythmia-related events discrimination) were found to be predictive, with an increase of 1% of the global native T1 Z-score leading to a + 27% higher risk of arrhythmia-related event occurrence (OR 1.27 [1.02–1.59], p = 0.035, AUC 0.69) (Table 2). Patients with ECV > 30.5% were at higher risk respectively of arrhythmia-related events, with an almost fourfold increase in risk when the value was above this cutoff (OR 3.58 ([1.21–10.61], p = 0.022, AUC 0.65) (Table 2).

**Fig. 4:**
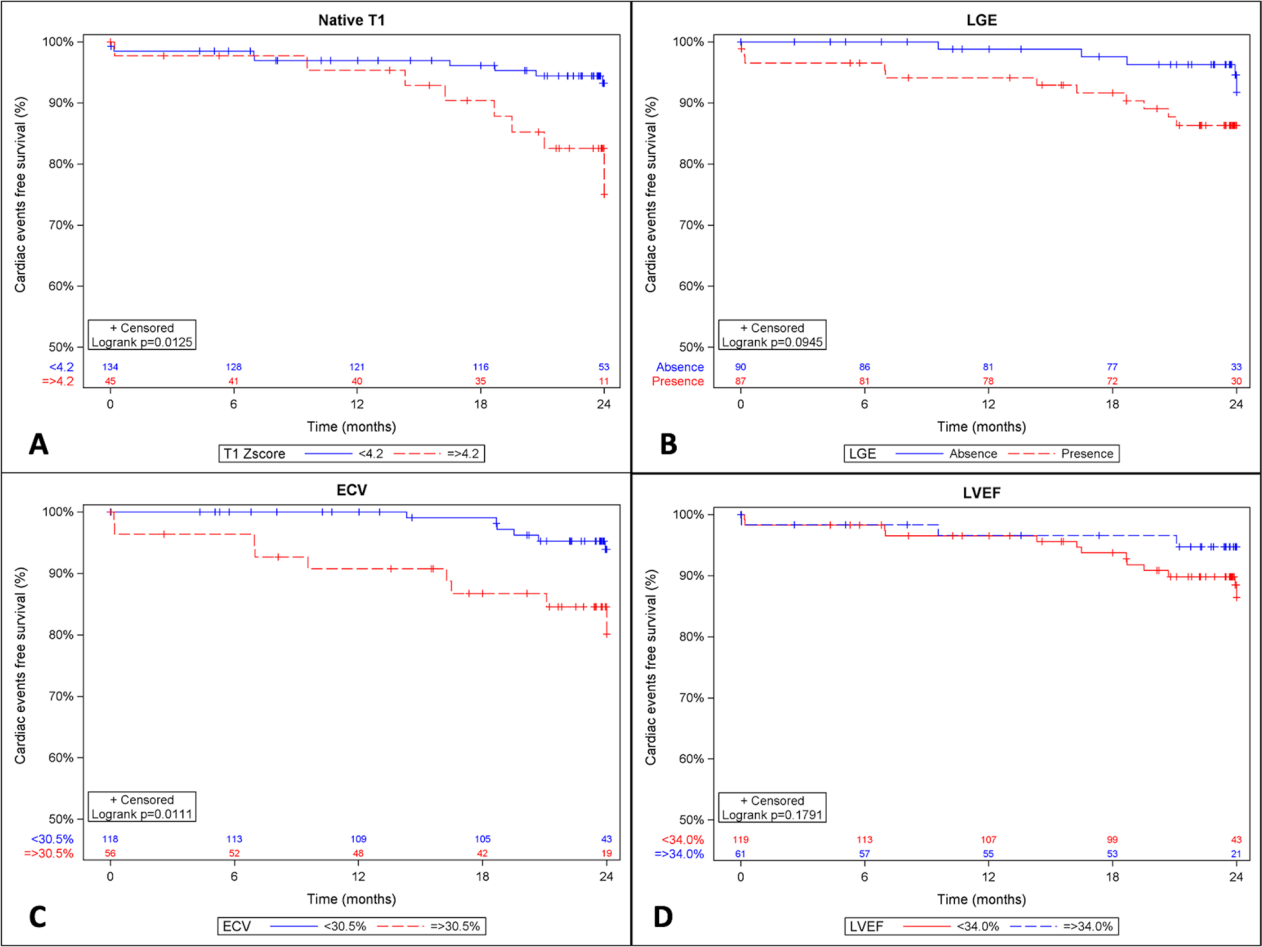
Cumulative 2-year arrhythmia-related event-free survival rate depending on CMR parameters. **A** Native T1 Z-score: < 4.2 vs. ≥ 4.2; based on the optimal cutoff determined by the Youden index. **B** Late gadolinium enhancement (LGE): present vs. absent. **C** Extracellular volume (ECV): < 30.5% vs. ≥ 30.5%; based on the optimal cutoff determined by the Youden index. **D** Left ventricular ejection fraction (LVEF): < 34.0% vs. ≥ 34.0%; based on the optimal cutoff determined by the Youden index

Kaplan–Meier curves showed that patients with either native T1 Z-score ≥ 4.2, or ECV ≥ 30.5% were at higher risk of arrhythmia-related events occurrence (p = 0.013 and p = 0.011 respectively) (Fig. 4). Multivariate analyses confirmed that ECV and native T1 Z-score above their respective optimal cutoff values (30.5% and 4.2 respectively) were the two CMR independent predictors of arrhythmia-related events occurrence (HR 2.72 [1.01–7.36], p = 0.049, and HR 2.86 [1.06–7.68], p = 0.037 respectively) (Table 3). Atrial fibrillation was also significantly associated with the prediction of arrhythmia-related events (p = 0.012).

## Discussion

Our study investigated the predictive value from quantitative CMR features for MACEs in NIDCM patients and reported the following main findings: (1) NIDCM patients with HF-related or arrhythmia-related events had higher native T1 and ECV values compared with NIDCM patients without MACEs; (2) noninvasive measures of diffuse interstitial fibrosis by native T1 and ECV were significantly predictive of MACEs; (3) increased ECV remained the only significant independent parameter predictive for both HF- and arrhythmia-related events; (4) increased native T1 (Z-score > 4.2) was also an independent predictor of arrhythmia-related events in NIDCM patients.

The two main complications for NIDCM patients are HF- and arrhythmia-related events. Traditionally, guidelines recommend ICD for primary prevention of SD based on NYHA and LVEF. However, it is precisely the patients who may not be eligible to ICD due to these guidelines that are likely to benefit from ICD thanks to their lower competing risk of non-SD. In this perspective, the latest European guidelines for the management of patients with ventricular arrhythmias and the prevention of SCD suggest (class of recommendation IIa) for the first time ICD implantation in NIDCM patients with LVEF > 35% (LVEF < 50%) and two or more risk factors [22]. Those risk factors include syncope, presence of LGE, inducible sustained monomorphic VT at programmed electrical stimulation, and high-risk genetic variants. Considering the LGE as a risk-factor [23, 24] but its inherent limitation to identify diffuse myocardial disease, our study investigated the predictive value of T1 and ECV in arrhythmia-related events in NIDCM patients as a primary outcome, which makes its originality*.* We found in our study that ECV was the sole independent predictor of both HF- and arrhythmia-related events with an almost 2 to threefold higher risk when above a cutoff of 32.1% and 30.5% respectively. By contrast, native T1 was only independently associated with arrhythmia-related events and might therefore be more useful to select patients eligible for ICD. Our results on ECV are consistent with the recent literature. Two recent cohorts analyzed T1 and ECV prognostic value in arrhythmia-related events in NIDCM patients [12, 25]. Both studies found that ECV was the strongest independent predictor of arrhythmia-related events with an ECV optimal cut-off value very similar to ours, strengthening the potential role of ECV as a predictive marker of arrhythmia-related events. By contrast, native T1 was not independently associated with arrhythmia-related events in those two studies. The differences in native T1 findings may come from differences in the cohorts, in the methodology to map T1, as well as differences in the number of arrhythmia-related events. Indeed, in Di Marco et al. [25], despite a larger cohort, there was a lower number of arrhythmia-related events, with only 2% of events compared to 7% in our cohort. Also, T1 was evaluated only in the mid-ventricular short axis slice. The prevalence of arrhythmia-related events also differed from the one found in Rubis et al. [12] who had different outcomes for arrhythmia burden endpoint (presence of VT or high burden of premature ventricular contraction). The consistency in ECV findings may suggest ECV consideration as an additional risk factor along those already included in the brand new 2022 European Society of Cardiology guidelines [22], while the place of native T1 remains to be further investigated.

Another interesting finding that we share with Rubis et al., is that replacement fibrosis assessed with LGE was not a significant predictor of arrhythmia-related events (p = 0.12) in NIDCM patients. Commonly, LGE is an accepted parameter for predicting cardiac outcomes, with midwall fibrosis being associated with MACEs [23, 24, 26]. Its importance has also been reconfirmed in Di Marco et al. study [25] in arrhythmia-related events in non-ischaemic cardiomyopathies. Nevertheless, this difference of LGE prognostic value in NIDCM patients may be due to the causes of DMC, to differences in the population baseline characteristics but also in LGE evaluation. In our study, almost half of patients without any MACE had an LGE, which is comparable to Rubis et al. population [12] but much higher than Di Marco et al. population. The high prevalence in our cohort of LGE in both MACE and without MACE group may explain the lack of prognostic value of LGE, but also suggests a limitation and lack of reproducibility of LGE assessment compared to quantitative tissue characterization based on T1 and ECV. Our study therefore supports the pathophysiological role of diffuse interstitial fibrosis in NIDCM and may offer perspectives in clinical management and early therapeutic intervention. In this regard, Di Marco et al. [25] therefore proposed a risk-model based on LVEF, LGE, and also ECV which achieved an excellent predictive ability for arrhythmia-related events in NIDCM patients. Finally, our findings could also raise awareness about the need for early detection of myocardial disease through T1 and ECV, prior to any NYHA or LVEF impairment, or LGE presence.

### Limitations

The main limitations of our study are the short-term follow-up and its relatively small sample size. These factors may explain the overall low rate of MACEs, especially in the arrhythmia-related events group which may limit the prognostic factors because of a lack of statistical power. Moreover, CMR referral itself introduced a selection bias, and our population might be more likely to be stable and to not have severe LVEF impairment given the exclusion of patients with an ICD. In addition, we did not perform quantitative evaluation of LGE because our study was focused on T1 and ECV. Finally, the etiology in DCM patients may impact the prognosis of CMR findings but was not known in our patients, to best match clinical practices; this point should be an interesting avenue for further studies.

## Conclusion

In patients with NIDCM, noninvasive assessment of myocardial fibrosis by quantitative ECV was predictive of both HF- and arrhythmia-related events. Native T1 (Z-score > 4.2) was also an independent predictor of arrhythmia-related events, which may therefore be useful for improved selection of patients for ICD. The addition of these quantitative CMR markers of diffuse interstitial fibrosis in the decision-making algorithm may improve arrhythmia risk stratification in NIDCM patients.

## Supplementary Information

**Additional file 1: Table S1.** CMR scans details on each site. **Table S2.** Typical CMR protocol details. **Table S3.** Comparisons between patients without major adverse cardiac events (MACE) and patients with the two subgroups of MACE. **Table S4.** Causes of heart failure (HF)-related (n = 42) and arrhythmia-related events (n = 16) in MACE (n = 58) patients. **Table S5.** CMR parameters—univariate analysis in the prediction of MACE. **Table S6.** Multivariate analysis of MACE prediction—stepwise Cox proportional hazards. **Figure S1.** Cumulative 2-year MACE-free survival rate depending on CMR parameters. (A) Native T1 Z-score: < 4.0 vs. ≥ 4.0; based on the optimal cutoff determined by the Youden index. (B) Late gadolinium enhancement (LGE): present vs. absent. (C) Extracellular volume (ECV): < 30.1% vs. ≥ 30.1%; based on the optimal cutoff determined by the Youden index. (D) Left ventricular ejection fraction (LVEF): < 27.8% vs. ≥ 27.8%; based on the optimal cutoff determined by the Youden index.

## Data Availability

All data generated or analyzed during this study are included in this published article and its Additional files.

## Abbreviations

CMR: Cardiovascular magnetic resonance
DCM: Dilated cardiomyopathy
ECG: Electrocardiogram
ECV: Extracellular volume
HF: Heart failure
ICD: Implantable cardioverter-defibrillator
LGE: Late gadolinium enhancement
LV: Left ventricle/left ventricular
LVEDV: Left ventricular end-diastolic volume
LVEF: Left ventricular ejection fraction
MACE: Major adverse cardiovascular event
MOLLI: Modified Look-Locker inversion recovery
NIDCM: Nonischemic dilated cardiomyopathy
NYHA: New York Heart Association
SD: Sudden death
VT: Ventricular tachycardia
