# Supplementary material for: Prognostic value of cardiovascular magnetic resonance T1 mapping and extracellular volume fraction in nonischemic dilated cardiomyopathy
Source: J Cardiovasc Magn Reson. 2023 Feb 6;25:7. doi: 10.1186/s12968-023-00919-y (PMC9900939; doi:10.1186/s12968-023-00919-y)
Supplement: Supplementary file 1 — Additional file 1: Table S1. CMR scans details on each site. Table S2. Typical CMR protocol details. Table S3. Comparisons between patients without major adverse cardiac events (MACE) and patients with the two subgroups of MACE. Table S4. Causes of heart failure (HF)-related (n = 42) and arrhythmia-related events (n = 16) in MACE (n = 58) patients. Table S5. CMR parameters—univariate analysis in the prediction of MACE. Table S6. Multivariate analysis of MACE prediction—stepwise Cox proportional hazards. Figure S1. Cumulative 2-year MACE-free survival rate depending on CMR parameters. (A) Native T1 Z-score: < 4.0 vs. ≥ 4.0; based on the optimal cutoff determined by the Youden index. (B) Late gadolinium enhancement (LGE): present vs. absent. (C) Extracellular volume (ECV): < 30.1% vs. ≥ 30.1%; based on the optimal cutoff determined by the Youden index. (D) Left ventricular ejection fraction (LVEF): < 27.8% vs. ≥ 27.8%; based on the optimal cutoff determined by the Youden index. [file 12968_2023_919_MOESM1_ESM.docx]

**ADDITIONAL FILE 1**

# Table S1. CMR scans details on each site.

|  | **Scanner** | **Vendor** | **Model** | **Coil** | **Gradient Strength**  (mT/m) | **Slew rate**  (T/m/s) | **Normal global native T1 values**  (mean ±SD, ms) |
| --- | --- | --- | --- | --- | --- | --- | --- |
| **1.5T** | 1 | Siemens | Avanto | Body | SQ45; Q33 | SQ200; Q125 | 980 ±35 |
|  | 2 | Siemens | Aera | Body | XJ33; XQ45 | XJ125; XQ200 | 1006 ±28 |
|  | 3 | Siemens | Avanto | Body | SQ 45; Q33 | SQ200; Q125 | 965 ±35 |
|  | 4 | Siemens | Avanto | Body | SQ45; Q33 | SQ200; Q125 | 950 ±30 |
|  | 5 | Siemens | Avanto | Heart | SQ 45; Q33 | SQ200; Q125 | 1150 ±30 |
|  | 6 | Siemens | Avanto | Body | SQ45; Q33 | SQ200; Q125 | 945 ±35 |
|  | 7 | Siemens | Avanto | Body | SQ45; Q33 | SQ200; Q125 | 956 ±28 |
|  | 8 | Siemens | Avanto | Body | SQ45; Q33 | SQ200; Q125 | 964 ±25 |
|  | 9 | Siemens | Symphony Tim | Body | 30 | 125 | 949 ±32 |
|  | 10 | Siemens | Aera | Body | XJ33; XQ45 | XJ125; XQ200 | 1004 ±26 |
|  | 11 | Siemens | Avanto | Body | SQ45; Q33 | SQ200; Q125 | 949 ±25 |
|  | 12 | Siemens | Avanto | Body | SQ45; Q33 | SQ200; Q125 | 995 ±27 |
|  | 13 | Siemens | Avanto | Body | SQ45; Q33 | SQ200; Q125 | 965 ±35 |
| **3T** | 1 | Siemens | Verio | Heart | 45 | 200 | 1177 ±37 |
|  | 2 | Siemens | Skyra | Body | 45 | 200 | 1220 ±35 |

| **Sequences** | **Type of sequence** | **Parameters** |
| --- | --- | --- |
| Cine | Balanced steady-state free precession sequence | slice thickness,7 mm; field of view, 320 × 320 mm2; temporal resolution, 28.9 ms; TE/TR, 1.6/3.6 ms; flip angle, 35◦; image  resolution, 256 × 144–222 |
| Late gadolinium enhancement | gradient echo inversion-recuperation sequence | TR/TE,650/1.56 ms; slice thickness, 6 mm; number of slabs, 14; flip angle, 10◦; field of  view, 350x400 mm^2^; matrix size, 152x134; and inversion time, 270–325 ms |
| T1 mapping pre contrast | 5 (3) 3 Modified Look-Locker Imaging | - At 1.5T: slice thickness, 6 mm; field of view, 360 ×360 mm2; TE/TR, 1.13/2.26   ms; flip angle, 35◦; matrix resolution,256  × 144–222   - At 3T: slice thickness, 6 mm; field of view, 360 mm2; TE/TR, 251.03/1.07 ms; flip angle, 35° |
| T1 mapping post contrast | 4 (1) 3 (1) 2 Modified Look-Locker Imaging |  |

**Demographics**

| Age, years | 57.5±14.5 | 58.3±13.7 | 55.5±16.6 | 0.348 |
| --- | --- | --- | --- | --- |
| Male, % | 146 (64.9) | 100 (61.7) | 46 (79.3) | **0.015** |
|  |  |  |  |  |

# All patients (n=225)

No HF- or Arrhythmia- related events **"No MACE" (n=167)**

HF- or Arrhythmia- related events **"MACE"**

# (n=58)

**p-value^†^**

|  |  |  |  |  |
| --- | --- | --- | --- | --- |
|  |  |  |  |  |
| Heart rate, beats/min *(nmiss=49)* | 73±16 | 72±16 | 76±17 | 0.052 |
| Systolic blood pressure, mmHg *(nmiss=43)* | 123±23 | 124±22 | 118±24 | **0.013** |
| Diastolic blood pressure, mmHg *(nmiss=43)* | 74±14 | 74±13 | 73±17 | 0.164 |
|  |  |  |  |  |
| NYHA functional class *(nmiss=43)* |  |  |  |  |
|  |  |  |  |  |
| ≤ II | 147 (80.8) | 115 (86.5) | 32 (62.3) | **0.002** |
| >II | 35 (19.2) | 18 (13.5) | 17 (34.7) | **0.002** |
| eGFR, ml/mn *(nmiss=32)* | 89±40 | 87±37 | 95±45 | 0.261 |
| Hematocrit, % *(nmiss=34)* | 41.9±4.5 | 41.5±4.4 | 42.9±4.5 | 0.110 |
| **Cardiovascular risk factors** |  |  |  |  |
|  |  |  |  |  |
| Body mass index, kg/m^2^ *(nmiss=31)* | 26.7±5.2 | 26.8±5.2 | 26.4±5.3 | 0.607 |
| Hypertension *(nmiss=28)* | 66 (33.5) | 52 (35.9) | 14 (26.9) | 0.305 |
| Diabetes mellitus *(nmiss=28)* | 29 (14.7) | 19 (13.10) | 10 (19.2) | 0.361 |
| Atrial fibrillation *(nmiss=28)* | 13 (6.6) | 6 (4.1) | 7 (13.5) | **0.044** |
| Dyslipidemia *(nmiss=28)* | 52 (26.4) | 40 (27.6) | 12 (23.1) | 0.586 |
| Smoking, current or previous *(nmiss=28)* | 76 (38.6) | 55 (37.9) | 21 (40.4) | 0.868 |
|  |  |  |  |  |
| Alcohol excess *(nmiss=30)* | 14 (7.2) | 10 (7.0) | 4 (7.7) | 1.000 |
| Family history of cardiomyopathy or SD *(nmiss=29)* | 32 (16.3) | 24 (16.7) | 8 (15.4) | 1.000 |
|  |  |  |  |  |

# Clinical and biological indices

RAS inhibitors *(nmiss=25)* 186 (93.0) 140 (95.2) 46 (86.8) 0.056

Diuretics *(nmiss=26)* 151 (75.9) 107 (73.3) 44 (83.0) 0.191

Beta blocker *(nmiss=26)* 183 (92.0) 136 (93.2) 47 (88.7) 0.376

# CMR parameters

| LVEF, % *(nmiss=3)* | 29.3±9.7 | 30.7±9.7 | 25.4±8.6 | **<0.001** |
| --- | --- | --- | --- | --- |
|  |  |  |  |  |
| LVEDV, ml/m^2^ *(nmiss=7)* | 145±48 | 141±47 | 157±48 | **0.013** |
|  |  |  |  |  |
| LV mass, g/m^2^ *(nmiss=10)* | 90.5±24.4 | 90.3±25.2 | 90.9±22.1 | 0.382 |
|  |  |  |  |  |
| LGE presence, % *(nmiss=7)* | 113 (51.8) | 76 (47.2) | 37 (64.9) | **0.030** |
|  |  |  |  |  |
| Native T1 Zscore *(nmiss=8)* | 3.0±2.3 | 2.7±2.2 | 3.8±2.3 | **0.001** |
| ECV, % *(nmiss=12)* | 29.3±4.1 | 28.7±3.8 | 31.1±4.3 | **0.001** |
| Global strain *(nmiss=29)* | -8.8±2.9 | -9.1±2.9 | -7.9±2.8 | **0.004** |

† Fisher's exact test for qualitative variables and Wilcoxon's test for quantitative variables. Reference group in "No MACE " (n=167). Values are mean ± standard deviation (SD), n (%). p≤0.05 (versus patients without MACEs)

HF= heart-failure, MACE= major adverse cardiovascular event, NYHA=New York Heart Association, eGFR=estimated glomerular filtration rate, SD=sudden death, RAS=renin-angiotensin-aldosterone system, CMR = cardiac magnetic resonance, LVEF= left ventricular ejection fraction, LVEDV= left ventricular end-diastolic volume, LV= left ventricular, LGE= late gadolinium enhancement, ECV= extracellular volume fraction, nmiss= number of missing values.

| Patients with MACE (n=58) | Type of event | Cause |
| --- | --- | --- |
| 1 | HF-related event | Hospitalization for acute HF |
| 2 | HF-related event | Hospitalization for acute HF |
| 3 | HF-related event | Hospitalization for acute HF |
| 4 | Arrhythmia-related event | Sudden death |
| 5 | HF-related event | Hospitalization for acute HF |
| 6 | HF-related event | Hospitalization for acute HF |
| 7 | Arrhythmia-related event | Sustained VT |
| 8 | HF-related event | Hospitalization for acute HF |
| 9 | HF-related event | Hospitalization for acute HF |
| 10 | HF-related event | Hospitalization for acute HF |
| 11 | Arrhythmia-related event | Sustained VT |
| 12 | Arrhythmia-related event | Appropriate ICD shock |
| 13 | Arrhythmia-related event | Sustained VT |
| 14 | Arrhythmia-related event | Appropriate ICD shock |
| 15 | HF-related event | Hospitalization for acute HF |
| 16 | Arrhythmia-related event | Sustained VT |
| 17 | HF-related event | Hospitalization for acute HF |
| 18 | HF-related event | Hospitalization for acute HF |
| 19 | HF-related event | Hospitalization for acute HF |
| 20 | HF-related event | Heart transplant |
| 21 | HF-related event | Heart transplant |
| 22 | HF-related event | Hospitalization for acute HF |
| 23 | HF-related event | Death due to cardiogenic shock |
| 24 | Arrhythmia-related event | Sustained VT |

| 25 | HF-related event | Hospitalization for acute HF |
| --- | --- | --- |
| 26 | Arrhythmia-related event | Appropriate ICD shock |
| 27 | HF-related event | Hospitalization for acute HF |
| 28 | HF-related event | Hospitalization for acute HF |
| 29 | HF-related event | Death due to cardiogenic shock |
| 30 | HF-related event | Hospitalization for acute HF |
| 31 | Arrhythmia-related event | Sudden death |
| 32 | HF-related event | Death due to refractory acute pulmonary edema |
| 33 | HF-related event | Heart transplant |
| 34 | HF-related event | Heart transplant |
| 35 | Arrhythmia-related event | Sustained VT |
| 36 | HF-related event | Hospitalization for acute HF |
| 37 | HF-related event | Heart transplant |
| 38 | HF-related event | Hospitalization for acute HF |
| 39 | HF-related event | Hospitalization for acute HF |
| 40 | HF-related event | Hospitalization for acute HF |
| 41 | HF-related event | Hospitalization for acute HF |
| 42 | HF-related event | Death due to refractory acute pulmonary edema |
| 43 | HF-related event | Death due to refractory acute pulmonary edema |
| 44 | HF-related event | Heart transplant |
| 45 | HF-related event | Hospitalization for acute HF |
| 46 | HF-related event | Hospitalization for acute HF |
| 47 | Arrhythmia-related event | Appropriate ICD shock |
| 48 | HF-related event | Death due to cardiogenic shock |
| 49 | Arrhythmia-related event | Appropriate ICD shock |
| 50 | HF-related event | Hospitalization for acute HF |
| 51 | Arrhythmia-related event | Sudden death |

| 52 | HF-related event | Hospitalization for acute HF |
| --- | --- | --- |
| 53 | HF-related event | Hospitalization for acute HF |
| 54 | HF-related event | Hospitalization for acute HF |
| 55 | Arrhythmia-related event | Sustained VT |
| 56 | HF-related event | Hospitalization for acute HF |
| 57 | HF-related event | Heart transplant |
| 58 | Arrhythmia-related event | Sustained VT |

HF= Heart failure; ICD= implantable cardioverter-defibrillator; MACE= major adverse cardiovascular event; VT= ventricular tachycardia

# MACE (n=58)

|  | **OR 95% CI^†^** | **p-**  **value** | **AUC^‡^** |
| --- | --- | --- | --- |
| HR | 1.02 1.00-1.04 | 0.089 | 59.5% |
| LVEF | 0.94 0.91-0.97 | **<0.001** | 66.7% |
| LVEF > optimal cutoff^††^ | 0.30 0.16-0.57 | **<0.001** | 64.5% |
| LVEDV | 1.01 1.00-1.01 | **0.038** | 61.1% |
| LV mass | 1.00 0.99-1.01 | 0.539 | 87.9% |
| LGE presence | 2.07 1.11-3.97 | **0.023** | 58.9% |
| Native T1 Zscore | 1.25 1.08-1.44 | **0.003** | 65.4% |
| Native T1 Zscore > optimal cutoff^‡‡^ | 3.54 1.86-6.76 | **<0.001** | 64.3% |
| ECV global | 1.16 1.07-1.26 | **<0.001** | 65.8% |
| Base ECV | 1.10 1.02-1.18 | **0.009** | 61.0% |
| Mid-ECV | 1.12 1.04-1.20 | **0.002** | 63.4% |
| Apex ECV | 1.09 1.01-1.16 | **0.024** | 58.8% |
| ECV > optimal cutoff^†††^ | 2.55 1.36-4.79 | **0.004** | 61.4% |
| ECV maximum | 1.09 1.04-1.15 | **0.001** | 62.5% |
| ECV ratio | 3.32 1.09-10.13 | **0.035** | 57.9% |

† Odds ratios with 95% confidence intervals (univariate logistic regression)

‡ Area under the ROC curve

†† The Youden index was used to depict optimal cutoff values from the ROC curves (27.8 for MACE, 27.8 for heart failure and 34.0 for arrhythmia)

‡‡ The Youden index was used to depict optimal cutoff values from the ROC curves (4.0 for MACE, 3.8 for heart failure and 4.2 for arrhythmia)

††† The Youden index was used to depict optimal cutoff values from the ROC curves (30.1 for MACE, 32.1 for heart failure and 30.5 for arrhythmia)

HF= heart failure, CMR= cardiovascular magnetic resonance, HR= heart rate, LVEF= left ventricular ejection fraction, LVEDV= left ventricular end-diastolic volume, LV= left ventricular, NYHA= New York Heart Association, LGE= late gadolinium enhancement, ECV= extracellular volume fraction.

**Outcome : MACE (n=225)**

**Adjusted**

**Variable**

**hazard- ratio**

**95% CI p**

| **Men (ref. Women)** | 2.35 | 1.23 | 4.50 | **0.010** |
| --- | --- | --- | --- | --- |
| **NYHA2>II (ref. ≤II)** | 2.59 | 1.43 | 4.71 | **0.002** |
| **LVEF>27.8^†^** | 0.50 | 0.28 | 0.88 | **0.016** |
| **ECV>32.1^†^** | 1.96 | 1.16 | 3.30 | **0.012** |

† For each outcome, the Youden index was used to depict optimal cutoff values from the ROC curves (see table 2)

HF= heart failure, LVEF= left ventricular ejection fraction, LVEDV= left ventricular end-diastolic volume, LV= left ventricular, NYHA= New York Heart Association, LGE= late gadolinium enhancement, ECV= extracellular volume fraction.

*p≤0.05 is considered significant.


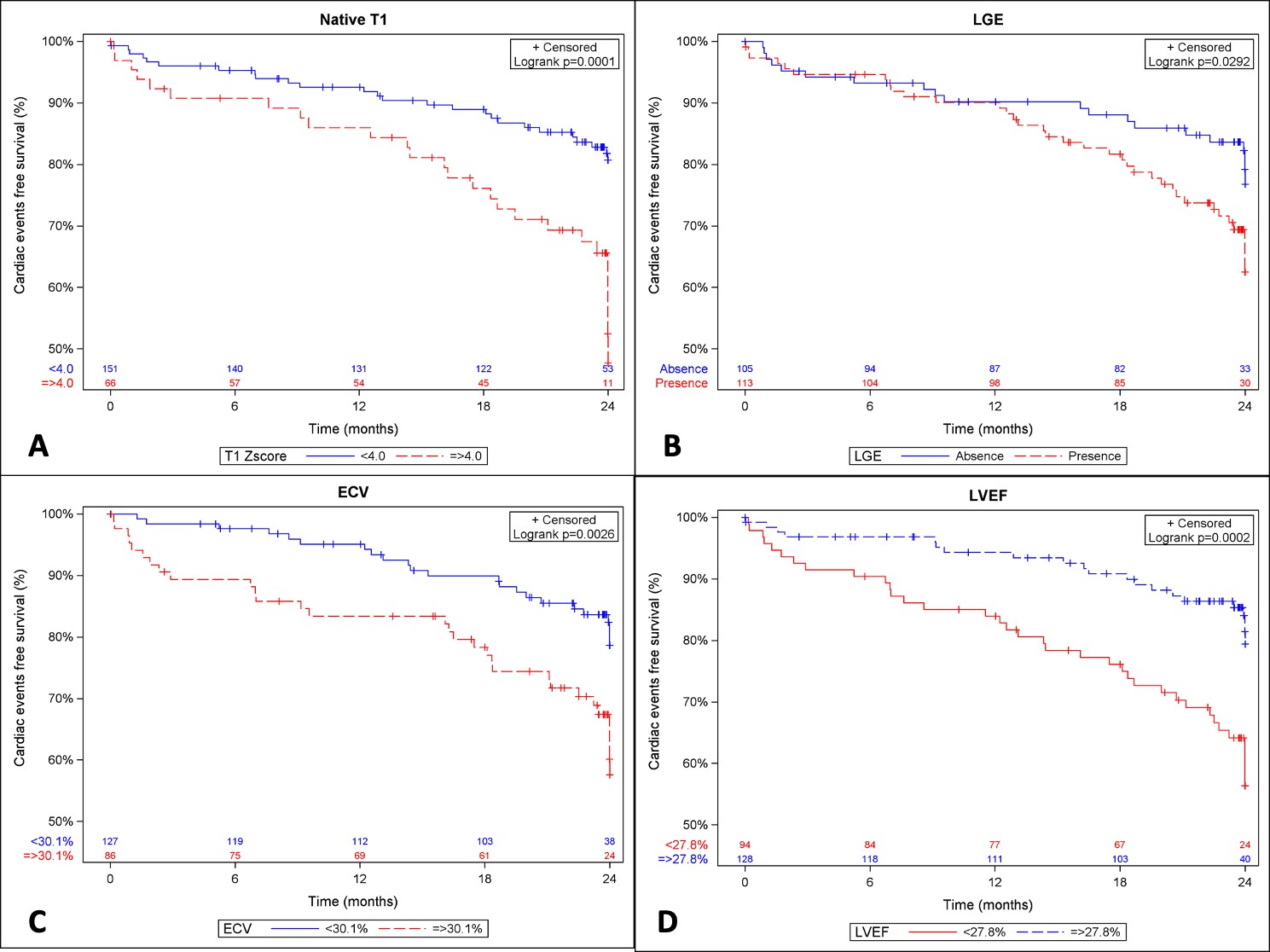


1. *Native T1 Z-score: <4.0 vs. ≥ 4.0; based on the optimal cutoff determined by the Youden index*
2. *Late gadolinium enhancement (LGE): present vs. absent*
3. *Extracellular volume (ECV): <30.1% vs. ≥ 30.1%; based on the optimal cutoff determined by the Youden index*
4. *Left ventricular ejection fraction (LVEF): <27.8% vs. ≥ 27.8%; based on the optimal cutoff determined by the Youden index*
